# Visualization and Quantification of APP Intracellular Domain-Mediated Nuclear Signaling by Bimolecular Fluorescence Complementation

**DOI:** 10.1371/journal.pone.0076094

**Published:** 2013-09-25

**Authors:** Florian Riese, Sonja Grinschgl, Manuel T. Gersbacher, Natalie Russi, Christoph Hock, Roger M. Nitsch, Uwe Konietzko

**Affiliations:** Division of Psychiatry Research and Psychogeriatric Medicine, University of Zurich, Zurich, Switzerland; Cleveland Clinic Foundation, United States of America

## Abstract

**Background:**

The amyloid precursor protein (APP) intracellular domain (AICD) is released from full-length APP upon sequential cleavage by either α- or β-secretase followed by γ-secretase. Together with the adaptor protein Fe65 and the histone acetyltransferase Tip60, AICD forms nuclear multiprotein complexes (AFT complexes) that function in transcriptional regulation.

**Objective:**

To develop a medium-throughput machine-based assay for visualization and quantification of AFT complex formation in cultured cells.

**Methods:**

We used cotransfection of bimolecular fluorescence complementation (BiFC) fusion constructs of APP and Tip60 for analysis of subcellular localization by confocal microscopy and quantification by flow cytometry (FC).

**Results:**

Our novel BiFC-constructs show a nuclear localization of AFT complexes that is identical to conventional fluorescence-tagged constructs. Production of the BiFC signal is dependent on the adaptor protein Fe65 resulting in fluorescence complementation only after Fe65-mediated nuclear translocation of AICD and interaction with Tip60. We applied the AFT-BiFC system to show that the Swedish APP familial Alzheimer’s disease mutation increases AFT complex formation, consistent with the notion that AICD mediated nuclear signaling mainly occurs following APP processing through the amyloidogenic β-secretase pathway. Next, we studied the impact of posttranslational modifications of AICD on AFT complex formation. Mutation of tyrosine 682 in the YENPTY motif of AICD to phenylalanine prevents phosphorylation resulting in increased nuclear AFT-BiFC signals. This is consistent with the negative impact of tyrosine phosphorylation on Fe65 binding to AICD. Finally, we studied the effect of oxidative stress. Our data shows that oxidative stress, at a level that also causes cell death, leads to a reduction in AFT-BiFC signals.

**Conclusion:**

We established a new method for visualization and FC quantification of the interaction between AICD, Fe65 and Tip60 in the nucleus based on BiFC. It enables flow cytometric analysis of AICD nuclear signaling and is characterized by scalability and low background fluorescence.

## Introduction

Even though recently modified, the leading hypothesis for the pathogenesis of Alzheimer’s disease (AD), the amyloid cascade hypothesis, assigns a pivotal role to Aβ [1-4]. Various forms of Aβ are released upon sequential cleavage of the amyloid precursor protein (APP) by the β-secretase BACE1 and the γ-secretase complex [5]. Another APP cleavage product, generated both through the amyloidogenic β-secretase-initiated and the non-amyloidogenic α-secretase-initiated pathway, is the APP intracellular domain (AICD). AICD forms transcriptionally active complexes with the multidomain adaptor protein Fe65 and the histone acetyltransferase Tip60 (AFT complexes) [6]. These complexes localize to distinct nuclear spots [7] that are sites of active transcription [8]. We could furthermore demonstrate that nuclear signaling capability of AICD is determined by the N-terminal residues that determine the propensity for proteasomal degradation [9]. Notably, formation of AFT complexes occurs predominantly after AICD release through the amyloidogenic processing pathway of APP [10]. Furthermore, many familial mutations that were found to be causative for AD were shown to have an effect on both Aβ and AICD production (e.g. the Swedish APP double mutation [10,11] as well as mutations mutations in the γ-secretase subunits presenilin 1 and 2 [12-14]). These and other observations indicate that AICD may have a role in the disease process alongside Aβ [15].

Bimolecular fluorescence complementation (BiFC) is a technique for visualization of protein-protein interactions [16-18]. It relies on the coupling of target proteins to fragments of fluorescent proteins, most commonly variants of yellow fluorescent protein (YFP). By themselves, these fragments are not fluorescent. However, upon interaction of the labeled target proteins, they are brought into proximity and complementation to a fully functional fluorescent protein occurs. The signal can then be detected by microscopy and BiFC-positive cells can be quantified by flow cytometry (FC) [19]. In the field of neurodegenerative diseases, BiFC has so far been used to study disease mechanisms in Alzheimer’s and Parkinson’s disease [20], such as the oligomerization of α-synuclein [21,22]. For APP, BiFC was employed to demonstrate the formation of APP homodimers in the endoplasmatic reticulum and Golgi apparatus and to study the differential dimerization properties of different isoforms and familial AD mutations of APP [23-25]. In another set of experiments, BiFC revealed the heterodimerization of APP with Notch2 [26,27]. Recently, BiFC was used to show the Mint2-mediated interaction of the APP C-terminus with Munc18 [28]. Finally, the interaction between Fe65 and another APP-interacting protein, LRP1, was demonstrated using BiFC [29].

In order to further study the regulation of AICD nuclear signaling we now developed a new method for visualization and quantification of AFT complex formation based on BiFC. This new method overcomes several limitations of the previously published method based on manual counting under the microscope [10], since it is feasible for FC analysis and therefore avoids inter-rater variability and allows a higher throughput.

## Methods

### Plasmid vectors

For generation of pUKBK-C-APP-YC155, the YC-BiFC fragment was PCR amplified from pBiFC-bFos-YC155 (courtesy of Tom Kerppola [30]) using primers agtcggcgcgccccgtccggcgtgcaaaatcc and tgcagtttaaacttacttgtacagctcgtccatgccg and cloned to the C-terminus of full length APP using the restriction enzymes AscI and PmeI, as described previously for the modular pUKBK vector system [31]. An alternative APP-YC155 construct featuring a shorter linker peptide between APP and the YC155 fragment, pUKBK-C-APP-YC155sl, was designed using primers agtcggcgcgcccatgaaccacgacaagcagaag and tgcagtttaaacttacttgtacagctcgtccatgccg and restriction enzymes AscI and PmeI. For generation of vector pUKBK-C-myc-Tip60-YN155 (with a C-terminal location of YN155), a YN155 fragment was produced by PCR with primers agtcggcgcgcccagatccatcgccaccatggtgag and tgcagtttaaacctaggccatgatatagacgttgtggctg on pBiFC-bJun-YN155 (courtesy of Tom Kerppola [30]). The resulting PCR product was then cloned into expression vector pUKBK-C-myc-Tip60 by restriction enzymes AscI and PmeI. For N-terminal localization of YN155 to Tip60, vector pUKBK-C-YN155-Tip60 was created following the same strategy but using primers cagttctagagctagcggccgcctcggccgccaccatggtgagcaaggg and cagttccggacaggtcctcctcgctgatcagcttctgctcggccatgatatagacgttgtgg and restriction cloning with XbaI and BspEI. Vector pUKBK-C-APP-YN155 was cloned from pUKBK-C-APP-YC155 and pUKBK-C-myc-Tip60-YN155 using restriction enzymes AscI and PmeI. Finally, pUKBK-C-SwAPP-YC155 harboring the Swedish mutation of APP (K595N/M596L) [32] and pUKBK-C-APP-YC155 Y682F were generated by site-directed mutagenesis. The expression vectors for APP-Citrine, CFP-Tip60, HA-Fe65 and HA-X11α were described previously [7].

### Cell culture

HEK293 cells (DSMZ) were cultured in DMEM (Gibco) supplemented with 10% fetal bovine serum (Invitrogen). For FC analysis, 400,000 HEK293 cells per well were seeded in 12-well plates and cultivated in 5% CO_2_ at 37°C. On the next day, transfections with equal vector amounts were performed using Lipofectamine 2000 (Invitrogen) following the manufacturer’s protocol. Three hours post transfection, medium was changed to DMEM/F12 supplemented with 25mM HEPES (Gibco). For oxidative stress experiments, H_2_O_2_ (Merck) was added at this point. On the following day, cells were kept in ambient air for 10 hours at 30°C to promote fluorescence maturation prior to FC analysis. For confocal imaging, 50,000 cells per well were seeded on 4-well object trays coated with poly-L-ornithine (50 µg/ml, Sigma) and fibronectin (5 µg/ml, Sigma). Transfections were performed as described above.

### Flow cytometry

For preparation of single cell suspensions, cells were washed with PBS (Gibco), trypsinized, pelleted, resuspended in PBS and strained through a nylon mesh cap (Falcon). Cell preparation and analysis were carried out consecutively for every sample alternating the experimental and control condition. Per sample, 150,000 cells were analyzed on a Cytomics FC500 (Beckman Coulter) with excitation at 488 nm and registration with the 525BP Fl1-filter. Cells were defined as BiFC positive if they fell in a rectangular gate that eliminates 99.9% of control condition cells (Lipofectamine 2000 alone).

### Western blotting

Cells remaining from the FC analysis were harvested and used for correction of possible variations in expression of the different APP constructs. Cells lysates were separated by SDS-PAGE on 10–20% tricine gels (Invitrogen) followed by Western blotting. Raw BiFC-FC cell counts were then normalized to the band-intensity ratio between APP (APP C-terminal antibody, Sigma, 1:4000) and βActin (Abcam, 1:1000) or GAPDH (Meridian Life science, 1:4000). Bands were visualized with HRP-coupled secondary antibodies (GE-Healthcare) and by ECL (Pierce), measured with the LAS-3000 camera system (Fujifilm Life Sciences) and analyzed using the Multi Gauge V3.0 software (Fujifilm Life Sciences).

### Immunocytochemistry

Immunocytochemistry was performed as described previously [7] using HA-antibody (Roche, 1:100) and Cy5-linked secondary antibody (Jackson, 1:250). To label subcellular compartments, anti-calnexin (Stressgen) and anti-TGN46 antibodies (Sigma) were used at a dilution of 1:100. DAPI (Sigma) was used to counterstain nuclei.

### Confocal microscopy

Images were acquired on a TCS/SP2 confocal microscope (Leica) as described previously [7].

## Results

### AFT-BiFC design

**Figure 1:**
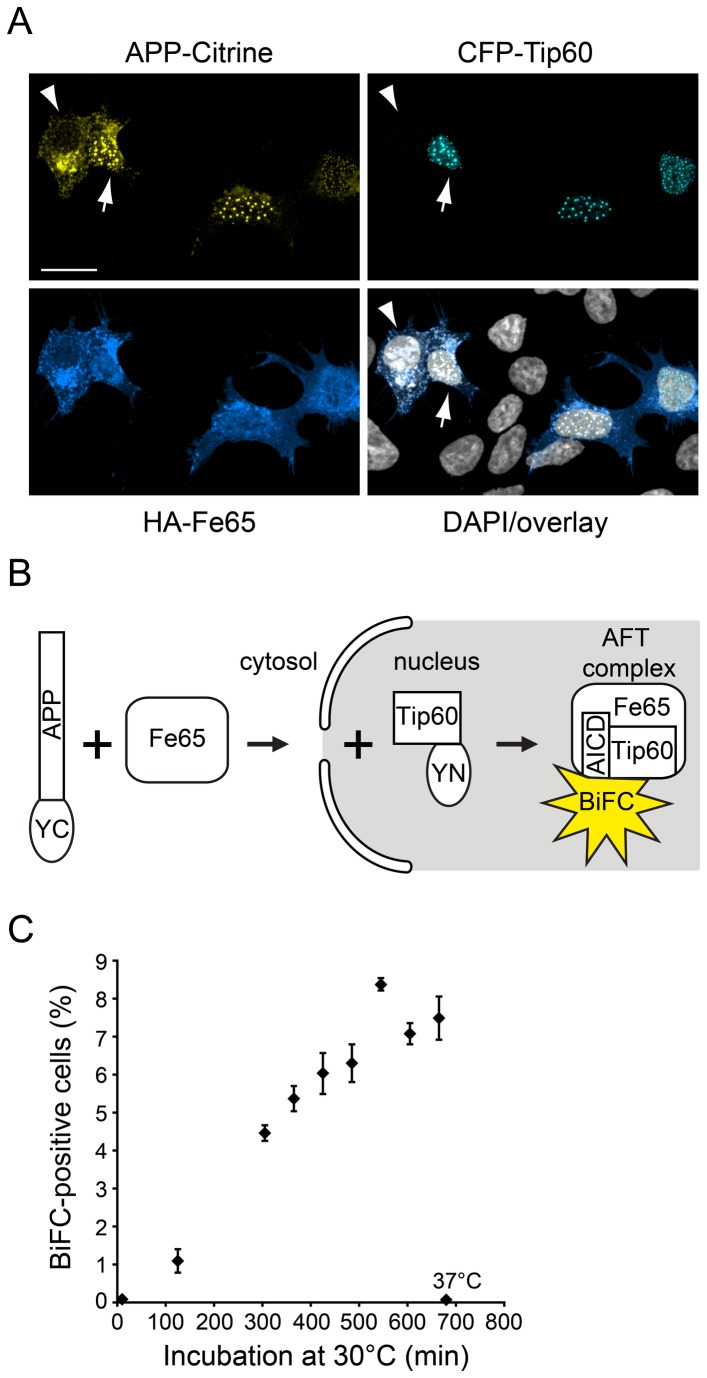
AFT complex formation and the BiFC principle. (A) Spherical nuclear AFT complexes in HEK293 cells cotransfected with APP-Citrine, CFP-Tip60 and HA-Fe65 (arrow). Cells that lack CFP-Tip60 accumulate neither AICD-Citrine nor HA-Fe65 in the nucleus (arrowhead). Nuclei were counterstained with DAPI. Length of bar: 20 µm. (B) Schematic depiction of the BiFC-based AFT complex detection system, where APP and Tip60 are fused to YFP halves. Since Fe65 serves as an adaptor between APP and Tip60, fluorescence complementation only occurs in the presence of all three proteins. (C) Fluorescence maturation in AFT-BiFC. Fluorescence maturation at 30°C was allowed for increasing time periods before FC quantification (n=3 per timepoint, error bars represent SEM). If samples were maintained at 37°C, no maturation occurred (samples labeled 37°C).

Classical fluorescence fusion constructs are suitable to demonstrate the subcellular localization of AFT complexes in nuclear spots using confocal microscopy (Figure 1A). However, flow cytometers measure total cellular fluorescence and cannot discern fluorescence of nuclear AFT complexes and APP-Citrine residing in the ER/Golgi apparatus. The recently developed fluorescence resonance energy transfer - fluorescence assisted cell sorting (FRET-FACS) might be a way to resolve this problem [33] as only two fluorescent proteins in close proximity will emit a FRET signal. We decided to base our assay on the BiFC technique that relies on splitting YFP into two halves and fusing them to the proteins under scrutiny. Reconstitution of a fully functional fluorescent protein occurs only when both BiFC-fusion proteins are in close proximity. We fused the YFP halves to the APP C-terminus and Tip60, ensuring that only upon nuclear translocation of AICD-Fe65 complexes and association with Tip60 a fluorescent signal will be generated (Figure 1B). Thus, a fluorescence signal should report bona fide AICD nuclear signaling. A crucial step in the design of BiFC constructs is the positioning of the BiFC fragments YN and YC relative to the labeled proteins [16]. In our case, the positioning of one half of YFP to the APP C-terminus was determined by our interest in studying AICD nuclear translocation. For localization with respect to Tip60, we tested both a YN-Tip60 and a Tip 60-YN construct. We furthermore tested two different APP-YC constructs that differed in their length of linker between APP and YC (data not shown). For optimal fluorescence yield and lowest background a pairing of APP-YC (with the longer linker consisting of 38 amino acids) and the Tip 60-YN (C-terminal positioning) were chosen. BiFC requires a maturation phase at 30°C to reconstitute YFP. We therefore performed a maturation time series to identify the timepoint when full maturation is reached, which was at 600 minutes (Figure 1C).

### Subcellular localization

**Figure 2:**
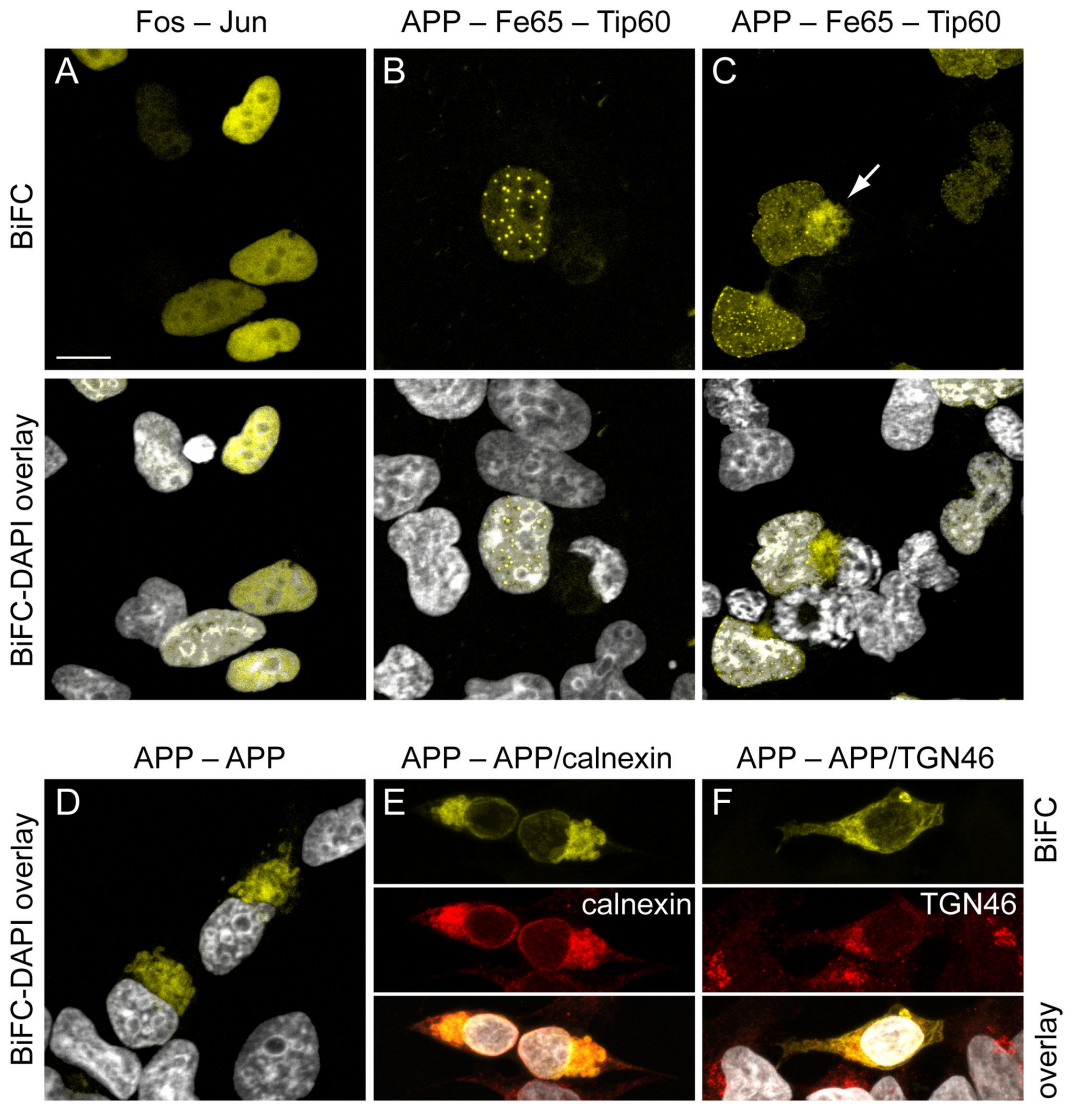
Subcellular localization of BiFC signals. HEK293 cells were imaged by confocal microscopy. (A) Cotransfection of bFos-YC and bJun-YN generates a BiFC signal distributed throughout the cell nucleus. (B) Cotransfection of APP-YC, HA-Fe65 and Tip 60-YN results in multiple spherical nuclear BiFC signals from AFT complexes. (C) In some cells, cotransfection also results in formation of extranuclear BiFC signals (arrow), which is consistent with an interaction of Tip60 and AICD/Fe65 also outside the nucleus as previously reported by us [8]. (D) Cotransfection with APP-YC and APP-YN results in perinuclear fluorescence (E+F). Colocalization of the APP/APP-BiFC signal with calnexin and TGN46 is consistent with the localization of APP/APP-dimers in the ER/Golgi. Nuclei were counterstained with DAPI. Length of bar: 13 µm (A, C, E, F) or 10 µm (B, D).

As previously described [30], bFos-YC and bJun-YN cotransfection results in an exclusively nuclear BiFC signal (Figure 2A). In contrast to this widespread nuclear localization, cotransfected APP-YC, Fe65 and Tip 60-YN show a nuclear spot-like distribution (Figure 2B) as known from cotransfection of classical full length fluorescent protein fusion constructs (Figure 1A). In some cases, AFT-BiFC could also be detected outside the nucleus (Figure 2C). Confocal analysis of the AFT-BiFC signal location revealed that in 56.4% of cells it was exclusively nuclear, 39.4% nuclear and extranuclear, and 4.2% were found to be exclusively extranuclear (n=312 fluorescent cells from nine confocal images). This means that AFT-BiFC has only a 4% error rate regarding fluorescent signals emanating from cells not harboring nuclear AFT complexes. In contrast to AFT-BiFC, cotransfection of APP-YC and APP-YN led to perinuclear BiFC, compatible with the known dimerization of APP (Figure 2D). The BiFC signal from APP dimers was found to colocalize with the endoplasmatic reticulum (Figure 2E) and the Golgi network (Figure 2F).

### AFT-BiFC flow cytometry

**Figure 3:**
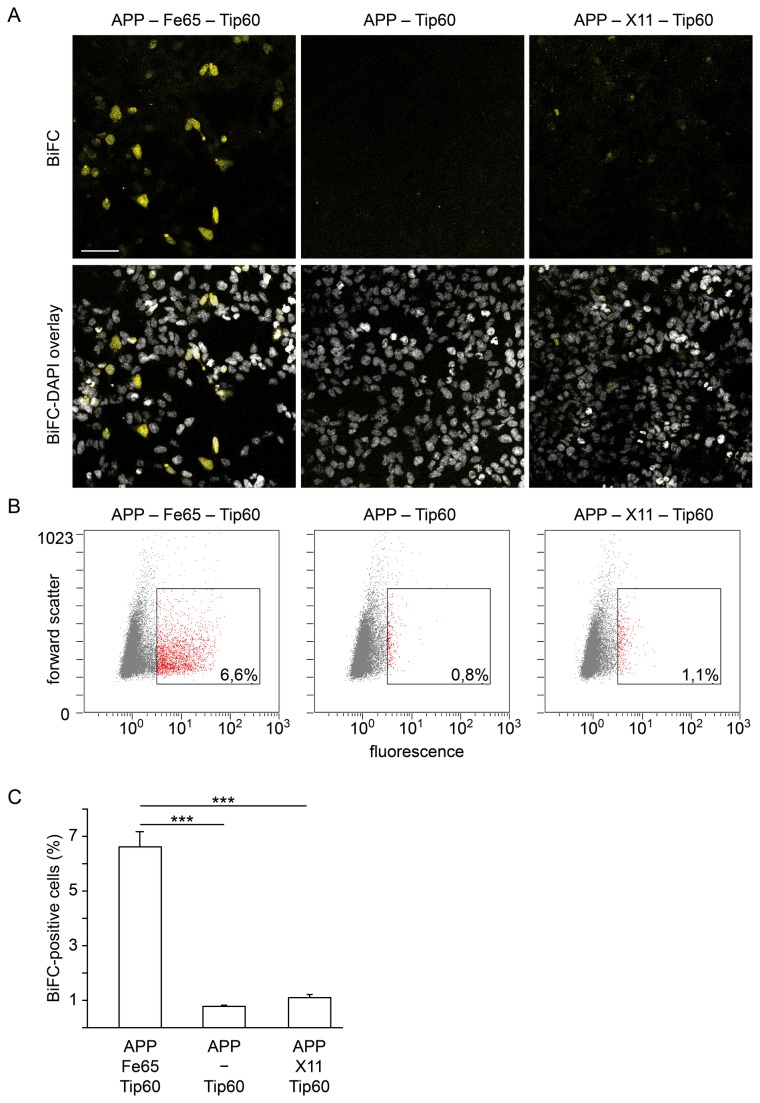
AFT-BiFC requires the presence of Fe65. (A) After cotransfection of APP-YC, Tip 60-YN and HA-Fe65 the BiFC signal can be detected in a subset of cells in the confocal microscope. In contrast, cotransfection of APP-YC and Tip 60-YN alone does not lead to fluorescence complementation, which indicates absence of direct interaction between AICD and Tip60. Transfection of APP-YC and Tip 60-YN together with the AICD-binding protein MINT1/X11 that traps AICD in the cytosol also does not generate a BiFC signal. Lower panels show BiFC overlay with DAPI nuclear staining. Length of bar: 60 µm. (B) Representative BiFC-FC scatter plots of individual samples. Percentages refer to gated cells. Fluorescence intensity and forward scatter are depicted in arbitrary units. (C) BiFC-FC quantification of HEK293 cells cotransfected with APP-YC and Tip 60-YN together with or without HA-Fe65 or with MINT1/X11 (n=6 vs. 5 vs. 6, error bars represent SEM, *** p<0.005, U-test). The approximately 1% BiFC-positive cells in the APP-Tip60 and APP-X11-Tip60 conditions are most likely due to background autofluorescence of HEK cells as well as fluorescence complementation mediated by endogenous Fe65.

Our AFT-BiFC system generates fluorescence only upon formation of AFT complexes, i.e. in the presence of all three interaction partners (Figure 3A). In the absence of Fe65 cotransfection, virtually no fluorescence is observed by microscopy or detected by FC (Figure 3 A–C). Likewise, replacement of Fe65 by another AICD-binding protein, HA-X11α, does not lead to a BiFC signal (Figure 3A) in line with HA-X11α trapping AICD outside of the nucleus [7]. Since background fluorescence was low and the signal was specific to AFT complex formation, we extended our system to quantification by FC. FC analysis verified our confocal results that Fe65 is required for production of a BiFC signal from APP-YC and Tip 60-YN and that this function is specific for Fe65 (Figure 3B and 3C). Identification of around 6% cells as BiFC-positive is consistent with our experience with classical fluorescent protein-coupled constructs (Figure 1A) generating approximately 5% cells with AFT complexes. We did not observe toxicity in cells harboring AFT complexes when measuring LDH release (data not shown). Additionally, we did not observe differences in the times required to reach a defined cell count during FC measurements between the tested conditions. We conclude that AFT-BiFC complex formation does not result in relevant cytotoxicity.

### Quantitative AFT-BiFC applications

**Figure 4:**
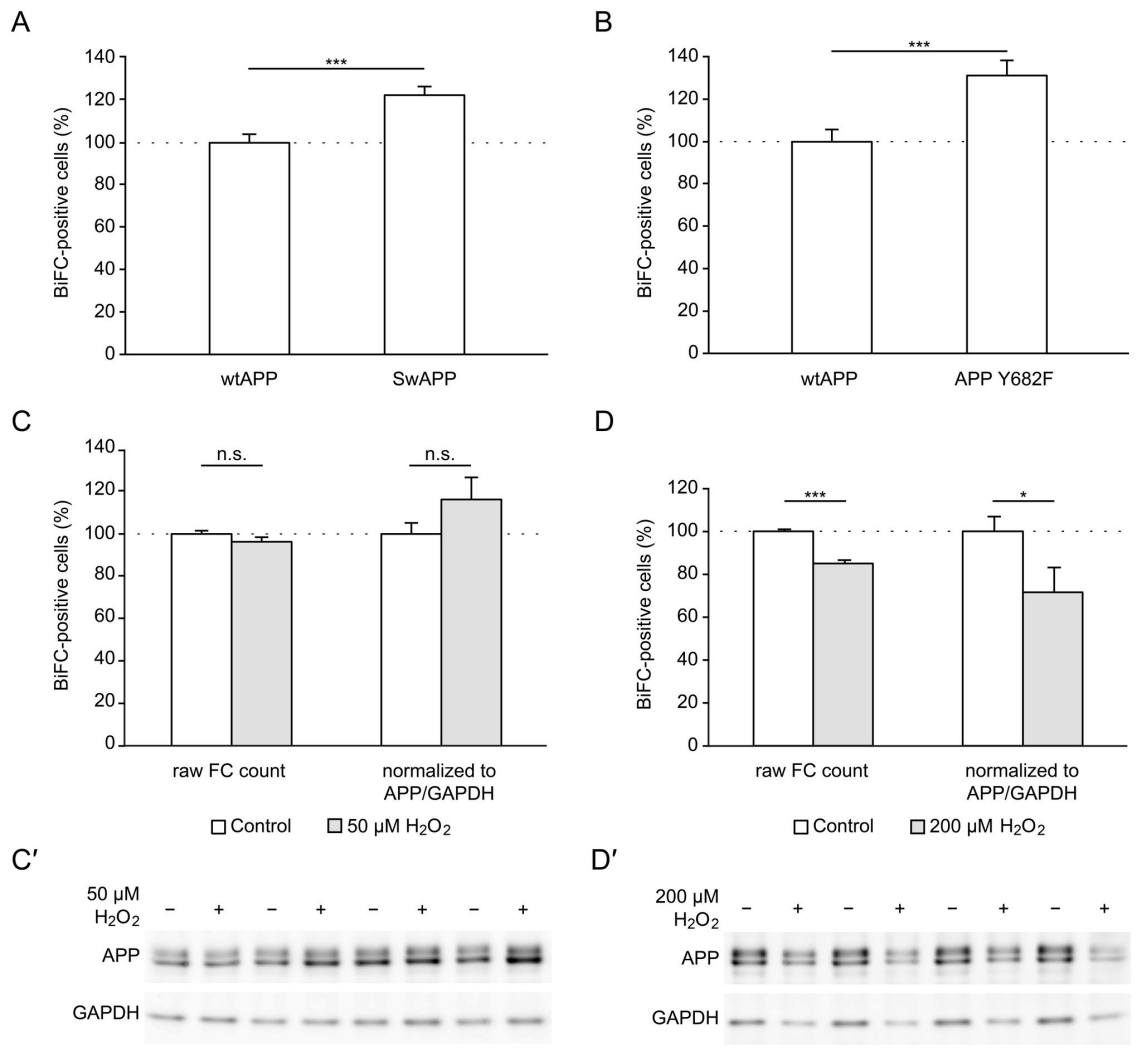
Quantitative AFT-BiFC applications. BiFC-FC quantification of HEK293 cells cotransfected with HA-Fe65, Tip 60-YN and different APP-YC constructs. (A) Swedish APP-YC increases nuclear AFT complex formation compared to wildtype APP (n=23 vs. 21 from two independent experiments, FC counts corrected for APP/βActin). (B) Mutation of tyrosine 682 to phenylalanine results in more AICD nuclear signaling (n=23 vs. 22 from two independent experiments, FC counts corrected for APP/βActin). (C) Oxidative challenge with 50 µM H_2_O_2_ does not affect nuclear AFT complex formation. Data were analyzed with or without correction for APP/GAPDH expression levels to correct for putative confounding toxic effects of H_2_O_2_ (n=22 vs. n=22 from two independent experiments). (C’) Representative Western blot of BiFC samples showing similar APP and GAPDH levels in both conditions indicating no toxic effect of H_2_O_2_ at this concentration. (D) Oxidative challenge with 200 µM H_2_O_2_ decreases nuclear AFT complex formation with or without correction for APP/GAPDH levels (n=20 vs. 20 from two independent experiments). (D’) Representative Western blot of BiFC samples showing decreased APP and GAPDH levels in the 200 µM H_2_O_2_ condition indicative of oxidative stress-induced cell death. For (A–D) Data were pooled from two independent experiments by setting the mean of wildtype APP-YC to 100%. Lipofectamine treatment alone (0.5% fluorescent cells) was used for gating. Error bars represent SEM, * p<0.05, *** p<0.001 (A, D) or p<0.005 (B), n.s. non-significant, t-test.

In order to provide proof-of-principle that quantification of AICD nuclear signaling with our BiFC-based method is possible not only for on/off-situations but also for more gradual differences, we compared wildtype APP-YC with Swedish APP-YC. The Swedish double mutation, which is known to cause a familial form of AD in humans [32], favors β-secretase cleavage of APP over α-secretase cleavage [11]. We previously showed that nuclear AICD in AFT complexes is predominantly generated through β-secretase activity and that the Swedish double mutation consequently increases AICD nuclear signaling [10]. Consistent with these findings, we measure a significant increase of AFT-BiFC positive cells with our novel system when comparing Swedish APP with wildtype APP (Figure 4A). Next, we were interested if BiFC can detect the influence of posttranslational modifications, specifically phosphorylation, on AICD nuclear signaling. The Y^682^ENPTY motif of AICD is essential for binding of many adaptor proteins including Fe65. Tyrosine 682 can be phosphorylated by several tyrosine kinases to differently affect the binding of adaptors [34]. We introduced the Y682F mutation in order to prevent phosphorylation at this position. In comparison to wildtype APP, this resulted in increased AICD nuclear signaling (Figure 4B). Finally, we tested whether challenging cell metabolism by oxidative stress alters nuclear AFT complex formation. We measured BiFC signals from cells expressing wildtype APP-YC with or without exposure to H_2_O_2_. At a concentration of 50 µM, we found no difference in the percentage of BiFC-positive cells with or without correction for APP and GAPDH levels (Figure 4C). Western blot analysis revealed similar GAPDH band intensities in both conditions (Figure 4C’) and total cell counts during FC were likewise similar indicating no overt cytotoxicity. At a H_2_O_2_ concentration of 200 µM, the percentage of BiFC-positive cells was reduced (Figure 4D). At this concentration, we observed lower APP and GAPDH band intensities on Western blots (Figure 4D’) and reduced total FC counts (data not shown) indicating H_2_O_2_-induced cell death. Nevertheless, the reduction in BiFC signal remained robust even after correction for APP and GAPDH levels (Figure 4D).

## Discussion

AICD functions as regulator of transcription for several genes [35]. Even though the precise mechanism remains to be elucidated, it likely involves the nuclear complex formation of AICD with Fe65 and the histone acetyltransferase Tip60, which are localized to the RNA polymerase II complex via the Mediator subunit MED12 [36]. Consistent with this notion, AFT complexes were found to localize to sites of active transcription [8]. Since the gene-regulatory function of AICD should itself be tightly regulated, we established a BiFC-based assay that allows the visualization of AFT complexes and their quantification by FC. With this new system, the regulatory effects of APP mutations, posttranslational APP modifications or general manipulations of cell metabolism on AICD nuclear signaling can be analyzed in a medium-throughput fashion.

In order to obtain BiFC constructs with high fluorescence yield and minimal background several plasmid variants were cloned. For APP a longer linker (38 amino acids) between its C-terminus and the YC-coding sequence was optimal, possibly because the interaction between AICD and Tip60 is indirect and mediated by Fe65. A hinge sequence may thus facilitate bringing the two BiFC fragments in sufficient proximity. Similarly, we found a stronger signal of the APP-YC/Tip60-YN pairing over APP-YC/YN-Tip60, indicating that in AFT complexes the AICD C-terminus is closer to the Tip60 C-terminus than to its N-terminus. In terms of subcellular localization of AFT complexes, our AFT-BiFC constructs demonstrate the same nuclear distribution in distinct spots as we have reported for AICD fusions to full-length fluorescent proteins and endogenous AICD [8,10]. This is clearly distinct from the homogenous nuclear distribution of bFos-YC/bJun-YN dimers [30]. Transcription has been described to occur in around 2000 nuclear loci called transcription factories [37,38]. Dimers of fos/jun are transcription factors that localize to transcription factories. We hypothesize that, due to the large number of genes reported to be regulated by fos/jun, microscopic resolution of single transcription factories in the microscope is probably not possible. In contrast, AFT complexes regulate a smaller number of genes thus enabling visualization of single sites.

In contrast to perinuclear BiFC signals arising from homodimers of APP in the ER/Golgi [23,24,26], AFT complexes predominantly localize to the nucleus. Nevertheless, in around 40% of the cells we also observed an AFT-BiFC signal originating from perinuclear sites. This is in line with our recent experiments showing a leptomycin B-dependent shuttling of Tip60 between nucleus and cytosol and a localization of AFT complexes to neuronal processes [8].

A major drawback of many BiFC applications is background fluorescence, i.e. fluorescence complementation in the absence of the condition that is expected to bring the labeled proteins into proximity [16]. With our system, virtually no background fluorescence is observed, most likely due to the indirect interaction between AICD-YC and Tip 60-YN that requires the adaptor protein Fe65 for fluorescence complementation. Since the assay depends on protein expression and fluorescence maturation, it does not allow for real-time imaging of AFT complex formation. In contrast to our previously published method for AFT complex quantification [10], the use of BiFC-FC that we report here does not rely on manual counting of AFT complex positive cells thus eliminating interrater variability. It also allows for a higher number of samples and number of cells per sample to be analyzed. Furthermore, with the possibility to subsequently normalize FC results to protein levels by Western blotting, variations in transfection efficiency can be easily controlled.

The validity of our novel BiFC assay for quantification is further underlined by the finding that the Swedish APP double mutation increases AFT nuclear signaling relative to wildtype APP. The Swedish double mutation increases APP cleavage by β-secretase, which was shown to be the predominant pathway leading to AICD nuclear signaling [10,39,40]. The relatively lower increase in AFT complex-positive cells (approx. 22%) that we measure with our assay when compared to manual counting (approx. 42%) [10] may point to a lower sensitivity of our system. This may at least in part be explained by the lower fluorescence intensity of EYFP-BiFC compared to full-length Citrine, which enables unequivocal identification of even weakly fluorescent nuclear AFT spots in the microscope. With our BiFC assay, we thus corroborate our previous finding that the Swedish APP double mutation increases AICD nuclear signaling [10]. We thereby provide further evidence that familial AD mutations also influence functions mediated by AICD.

Posttranslational modifications sculpt the function of proteins. Tyrosine 682 in the Y^682^ENPTY motif is crucial for the binding of Fe65 and other adaptor proteins. Phosphorylation of this residue by src, abl, trkA, and EGFR tyrosine kinases was reported to enhance the binding of SH2-domain containing adaptor proteins while abolishing binding of Fe65 [34,41-44]. Consequently, our BiFC assay revealed increased nuclear signaling when Tyrosine 682 was mutated to phenylalanine to prevent phosphorylation. Tyrosine 682 therefore seems to be phosphorylated in HEK293 cells resulting in diminished AICD nuclear signaling. We conclude that signals such as NGF, acting via trkA receptors [45], prevent AICD-Fe65 interaction and thus inhibit AICD nuclear signaling, whereas tyrosine phosphatases should increase signaling.

Elevated markers of oxidative damage are found in affected brain areas in AD [46]. Using our BiFC assay we could show that increasing oxidative stress to a point that results in cell death also diminishes AICD nuclear signaling. However, our data does not allow us to discern whether reduced AICD nuclear signaling under conditions of oxidative stress is causative for cell death or if both, reduced AICD signaling and cell death, are parallel consequences of oxidative stress.

In conclusion, we report here a novel BiFC-based assay for visualization and quantification of AICD nuclear signaling through AFT complexes. The assay is characterized by very low background and demonstrates the expected nuclear distribution of AFT complexes. It is suitable for FC analysis and therefore allows for medium-throughput quantification of the effects of familial APP mutations, posttranslational modifications or drugs on AFT complex formation.
